# Technology and museum visitor experiences: a four stage model of evolution

**DOI:** 10.1007/s40558-023-00252-1

**Published:** 2023-06-01

**Authors:** Siqi Emily Lu, Brent Moyle, Sacha Reid, Elaine Yang, Biqiang Liu

**Affiliations:** Department of Tourism, Sport and Hotel Management, Griffith University, Brisbane, QLD Australia

**Keywords:** Museum, Visitor, Experiences, Information and Communication Technology, Concepts, Evolution

## Abstract

Research on technology and museum visitor experiences has experienced exponential growth. Despite this, limited studies explicitly examine existing progress in research on the intersection between technology and museum visitor experiences. Specifically, there is limited understanding of how topics studied, and the concepts, theories, models, and frameworks embedded within have evolved in congruence with the forms and types of technology integrated into museum research across time. Consequently, this paper applied a systematic quantitative approach to assess trends in research on technology and museum visitor experiences by critically examining 122 studies. Findings revealed a clear shift of the concepts studied, with early literature focused on basic concepts such as learning and interaction with technology. As the body of knowledge matured, other concepts such as intention and behaviour emerged in discourse, with contemporary literature exploring satisfaction, enjoyment, and virtual presence. Despite this, limited consistency in theories, models, and frameworks applied across time, which reflected a stagnation in stimulating critical discussions in the existing discourse. Furthermore, the forms and types of technology used in studies on museum visitor experiences have shifted from basic computer displays, through to innovative smart technology. This research provides the first attempt to holistically classify and synthesise the evolution of research on technology designed to enhance museum visitor experiences. Five types of technology in the museum sector and a Four Stage Model of Evolution consisting of (I) ICT Incubation; (II) Smart Technology Adoption; (III) ICT Transformation; and (IV) Futuristic Innovation were proposed, which demarcates the evolution of the body of knowledge.

## Introduction

Throughout history museums have become engrained in contemporary society as the collectors, preservers, and custodians of historically important artefacts (Ambrose and Paine 2012; Brown and Mairesse 2018; Huang et al. 2022). This includes, though is not limited to, the preservation of unique and charismatic tangible and intangible culture and heritage, artefacts which are deemed valuable for humanity, and the conservation of relics of the natural environment for educational purposes (Barron and Leask 2017). Traditional scholarship on museums, commonly termed “Old Museology” (McCall and Gray 2014) has a tendency to focus on methods of curation for the elites of society (Vergo 1989). However, over time, Vergo (1989) coined the term “New Museology” in response to demographic and generational change in society, which reflected a desire to enhance the visitor experiences in order to promote wider societal interest in the custodianship of historically significant artefacts. Concomitantly, to address falling admissions rates and financial imperatives, scholarship began to challenge the traditional role of museums as collection-based institutions only catering to particular social groups such as cultural elites (He et al. 2018; Zhang et al. 2023). Museums have accelerated the desire to embrace a wider range of visitors, with discourse emerging on how to engage traditionally marginalised sectors of society by enhancing the visitor experiences (Slak and Mura 2023).

Fast forward to modern times, contemporary discourse on museums has taken an active role to embrace a wider audience in what Macdonald (2006, p 362) affectionately terms *“the turn to the visitor”*. Within this emerging body of knowledge, the role of technology in enhancing the visitor experiences has been rapidly expanding (Hughes and Moscardo 2017; Lee et al. 2020). Existing scholarship has focused on the critical role of technology in improving museum visitor experiences, with discourse predominantly centred on how technology can be leveraged to create memorable experiences for museum visitors (Yang and Zhang 2022), how different generations accept the integration of technology in museums (Kang et al. 2018; Traboulsi et al. 2018) and how to deliver museum visitor experiences online (Mason et al. 2021). This emphasis has left the understanding of the types and forms of technology which have been integrated to assess museum visitor experiences underexplored and conceptually underdeveloped. Understanding studies on how the types of technology utilised in research on museum visitor experiences have evolved through a systematic analysis of trends of publication volume in discourse, journal concentrations, geographic locations of the research, core concepts, theories, models, and frameworks, methods and data collection techniques applied, is a clear need. This urgency is exacerbated due to the advent of pressures for museums to pivot online as a consequence of the COVID-19 pandemic (O’Hagan 2021; Resta et al. 2021; Shang et al. 2023; Williams et al. 2015).

Subsequently the purpose of this manuscript is to critically assess scholarship focused on technology and museum visitor experiences (TMVEs). By systematically tracking progress in existing studies, this manuscript develops four research objectives (RO):

- RO1: To identify the trends of publication volume, journals, and the geographic locations of research on TMVEs;
- RO2: To assess the evolution of concepts, theories, models, and frameworks utilised in research on TMVEs;
- RO3: To critically examine the types and forms of technology used in research on TMVEs;
- RO4: To explore progress in methods and data collection techniques applied in research on TMVEs.

This manuscript contributes to the literature on museum visitor experiences through the synthesis of the content above to develop a Four Stage Model of Evolution, which depicts the integration of technology in the museum context across time, explicitly classifying the different forms and types of technology utilised in museums and examining how topics studied have shifted. This manuscript contributes to the conceptual classification of the efficacy of technology for enhancing visitor experiences in museums, unearthing an evolution in research foci across time depicted in a Four Stage Model of Evolution. Practically, the conceptual model serves as a guide for museum practitioners to understand the trends of existing application of technology and its effectiveness and consider the future integration of technology in museums based on the fourth stage of the model (Neuhofer et al. 2014).

## Methods

Recent years have seen an increase in the use of systematic quantitative literature review (SQLR) in tourism research (Yang et al. 2020). Although the approach has been criticised for being descriptive, supporters note the SQLR provides a comprehensive, transparent, and reproducible approach for mapping the extant literature and laying the foundation for future research (Pickering and Byrne 2014). This review is particularly suited to unpacking an emerging or fragmented topic (Yung and Khoo-Lattimore 2019), which is yet to be adequately synthesised, such as TMVEs. With the growth of ICT studies in tourism, an examination of prior research is critical and timely to critically examine how technology applied in research has evolved, and the subsequent impacts on museum visitor experiences, providing a foundation designed to stimulate constructive discourse on the subject.

**Fig. 1:**
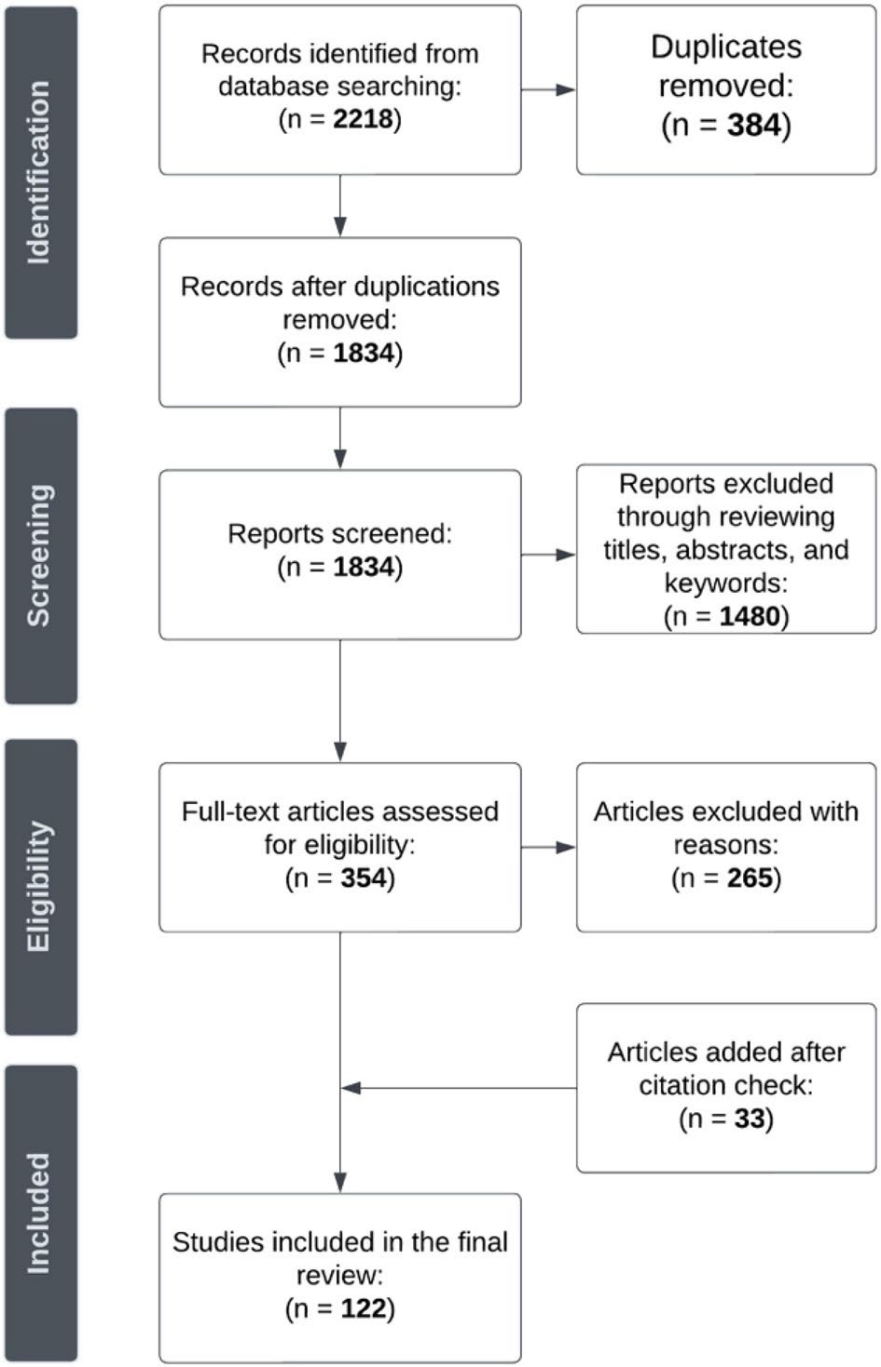
PRISMA

The Preferred Reporting Items for Systematic Literature Reviews and Meta-Analysis (PRISMA) has been extensively used in conceptually related studies which conduct a systematic review (Le et al. 2019). This research follows PRISMA for its transparent step-by-step procedure (Moher et al. 2009) (see Fig. 1).

A combination of 11 keywords was selected, including three categories Term A, Term B, and Term C, an approach followed by Chang et al. (2022), Table 1 below depicts three groups of keywords, which were selected from conceptually related studies, with the final words selected determined through a process of expert review by consulting with relevant experts in smart tourism and museum fields (Hadinejad et al. 2019). Term A was selected from the context of this study museum. Term B was applied, as it is a recurrent keyword in relevant studies and reflects the visitor experience component of the analysis (Roppola 2012). Terms in column C was identified and applied from conceptually related literature reviews on technology (see examples Shafiee et al. 2021; Yung and Khoo-Lattimore 2019).

**Table 1:** Keywords selected for search

Term A	Term B	Term C
museum*	experience*	technolog*
		information and communication technolog* (ICT*)
		Smart
		mobile
		Digital
		mixed realit*
		virtual realit*
		augmented realit*
		extended realit*
		Internet of Thing* (IoT)

Following a process articulated by Law et al. (2014) and Yang et al. (2017) five databases including Scopus, Web of Science, Emerald Insight, ScienceDirect, and EBSCOHost (Hospitality & Tourism Complete) were selected for the initial search, and Google Scholar was added as a quality control mechanism to ensure articles were not overlooked. Google Scholar is considered a sufficient source and acts as a reinforcement for including relevant articles (Le et al. 2019). As of 30th November 2022, the search was conducted, and articles published after the date were not given consideration. The initial search generated 2218 records, and after duplicates were removed, 1834 records were screened. Only English peer-reviewed journal articles were selected to safeguard the quality and consistency of the review, and it is a regular occurrence in SQLR studies (Le et al. 2019). After titles, abstracts, and keywords were checked, it excluded 1480 reports. 354 articles were assessed in full text, with 265 articles excluded for reasons. Table 2 summarises the inclusion and exclusion criteria applied to guide the analysis. The most common reasons of exclusion were that studies were not explicitly relevant to research on TMVEs. For instance, studies attempted to build a digital transformation assessment at an organisational level to help curators and professionals enhance visitor experiences, which is not explicitly focused on TMVEs (Agostino and Costantini 2022). In addition, another body of work excluded articles which examined technologies with a core emphasis on the system design and evaluation of technology (Ferrato et al. 2022), which deviates from the study focus.

**Table 2:** Inclusion and exclusion criteria

Criteria	Inclusion	Exclusion
Study Type	Peer-reviewed journal articles	Books; book chapters; conference proceedings; working papers; theses and dissertations; research/government reports
Language	English	Non-English papers
Relevance	Explicit focus on TMVEs	Technology is only mentioned as an example rather than the core focus of the research
		Technology is the focus, but studies not explicitly connected to the visitor experience
		Focus on the evaluation of the installation of technology in museums, not on visitors
		Museum is not the context of the study

Following a process by Shafiee et al. (2021) and Yang et al. (2017) an additional reference list check of included articles in the database was conducted to identify potential missing studies, and as a result a further 33 articles were added in the review. As a result, 122 eligible articles were considered in the final synthesis and were entered into a Microsoft Excel spreadsheet for quantitative coding analysis. Examined items were the number of studies conducted, the geographic locations of research, journals, types of technology utilised, concepts, theories, models, and frameworks, methods and data collection techniques adopted for the purpose of this research. To minimise the author’s limitation on linguistic background and interpretation as a non-native English speaker, the author followed the approach by Le et al. (2019) that first coding 10% of articles, then 10% until all reviewed articles were assessed, and checked with the rest authors who are native speakers to safeguard the quality and consistency of the coding process.

## Results

**Fig. 2:**
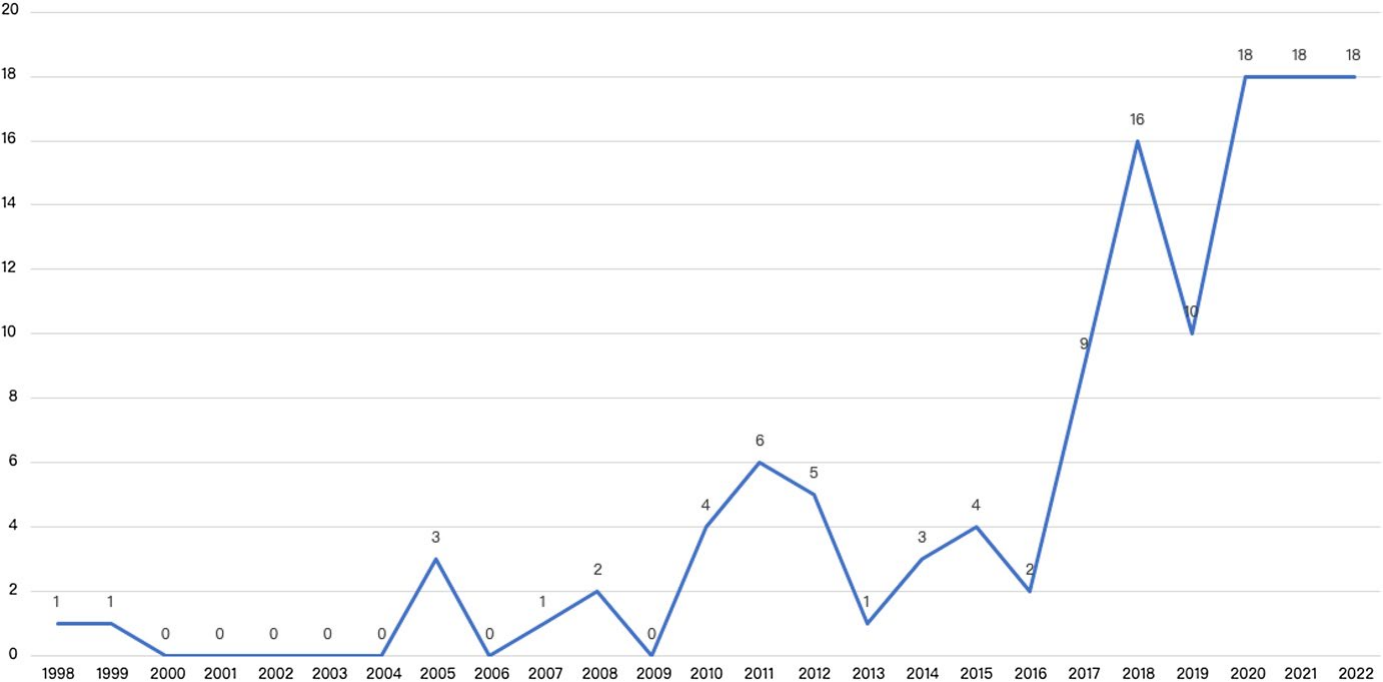
Growth in Research on TMVEs

Findings demonstrated an exponential growth of research on TMVEs particularly in the last five years (see Fig. 2). The earliest entry identified in the database was published in 1998, with research gaining traction slowly, with one article published the following year. Following the inception of these two early basic and descriptive studies, discourse on museum visitor experiences stagnated until 2005, when three manuscripts were published. Following 2006, discourse on TMVEs began to gain momentum, with sporadic growth experienced between 2005 and 2010. Momentum and constructive debate on TMVEs began to emerge in prominence in 2011 with 21 studies completed across the next six years. However, following 2016 evidence indicated a veritable explosion in research on TMVEs, with 89 studies conducted between 2017 and 2022.

Even though exponential growth in research on TMVEs commenced in 2017, the concentration of knowledge across journals was fragmented, with discourse spread across 68 peer-reviewed journals (an average of 1.79 papers per journal). It seems surprising to notice the absence of high impactful journal outlets, such as *Tourism Management*, *Annals of Tourism Research* and *Journal of Travel Research*, to name a few, with exception of He et al. (2018) in *Tourism Management*. This might be explained by the narrow understanding of what the museum is for and can do as an elitist place less attractive to the general tourist markets (Richards 2020). It is also evident that the knowledge concentration in *Museum Management and Curatorship* is museum specialised. Table 3 displayed the top six journals, which held 32.79% of research on TMVEs, with the remaining 62 journals including three articles or less.

**Table 3:** Key journals on TMVEs

Journals	No. of publications	%
*Museum Management and Curatorship*	11	9.02
*Current Issues in Tourism*	9	7.38
*Visitor Studies*	6	4.92
*ACM Journal on Computing and Cultural Heritage*	6	4.92
*Tourism Management Perspectives*	4	3.28
*Curator*	4	3.28
Total	40	32.79

**Fig. 3:**
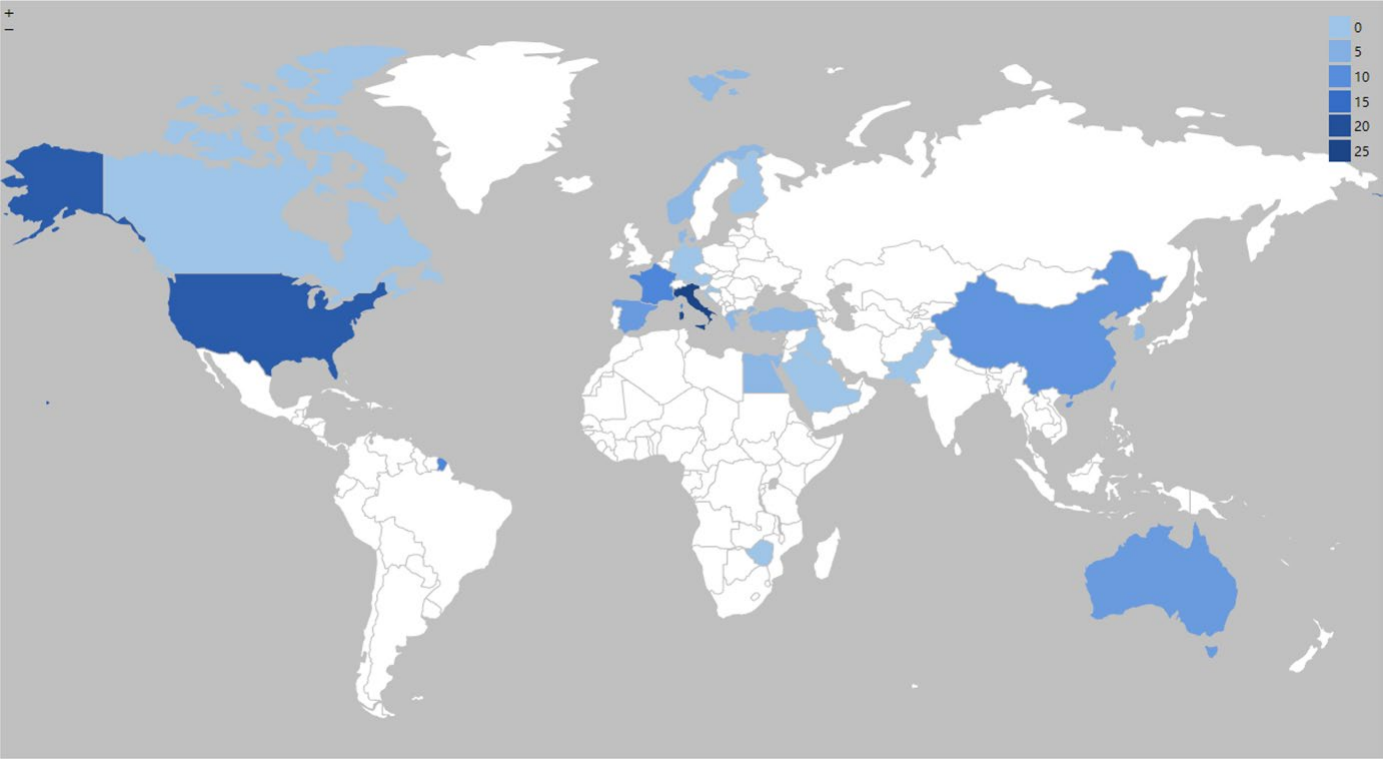
Geographic distribution of fieldwork locations

Regarding the distribution of geographic locations in research on TMVEs, studies were predominantly concentrated in Europe (73, 59.84%), Asia (25, 20.49%), and North America (20, 16.39%). In terms of specific countries, fieldwork was clustered in countries with a high concentration of museums, such as Italy (21, 17.21%), the United Kingdom (20, 16.39%), the United States (17, 13.93%), China (13, 10.66%), and France (10, 8.20%). While Europe enjoyed the largest number of fieldwork conducted in existing studies, the number of museums was ranked highest by the United Stated according to the latest statistics available (Statista 2021). Figure 3 visually presented a geographic map of fieldwork locations.

Table 4 presented the top ten concepts discussed in studies on TMVEs across time. The concepts were generated according to the title, abstract and keywords of each reviewed article, as they reflect central ideas of a paper (Moher et al. 2009). Accordingly, the first author extracted (for manifest), interpreted (for latent) core concepts of each article in the synthesis (Moyle et al. 2020). Interaction (31, 17.51%), experience (31, 17.51%), and learning (24, 13.56%) were the three most mentioned concepts in discourse. The concepts of interaction and experience began to gain scholarly attention in 2005. In 2011, other emergent concepts, such as intention, satisfaction, behaviour, and perceived ease of use complemented established studies on interaction and experience. The body of work experienced a shift in the concepts studied in 2017, with studies on intention, satisfaction, virtual presence, and enjoyment sparking an upsurge in research foci on TMVEs.

**Table 4:** Key concepts discussed over time

Core concepts	Frequency	1998–2004	2005–2010	2011–2016	2017–2022
Interaction	31		6	6	19
Experience	31		8	6	17
Learning	24	1	2	4	17
Intention	22			4	18
Satisfaction	14			2	12
Perceived Ease of Use	12		1	3	8
Virtual presence	12		1	1	10
Enjoyment	12		1		11
Behaviour	11			6	5
Attention	8		2	1	5
Total	177				

In early scholarship on TMVEs, studies were predominantly descriptive with limited theoretical engagement. Between 2011 and 2016, there was a gradual increase of theories, models, and frameworks applied in research, with seven articles applying theories, models, and frameworks. From 2017 an escalation of articles which applied theories, models and frameworks occurred, demonstrating research on TMVEs is maturing. However, atheoretical and descriptive studies still dominated existing discourse (77, 63.11%) (Table 5).

**Table 5:** Theories, models, and frameworks change across time

Theories/Models/Frameworks	No. of articles			
	1998–2004	2005–2010	2011–2016	2017–2022
Yes	0	2	7	36
No	2	8	14	53

Table 6 displayed the top three frequently applied theories, models, and frameworks in 122 TMVEs studies. 36 out of 122 studies adopted a single theoretical framework while nine articles utilised two or more theories, models, and frameworks. The Technology Acceptance Model (TAM) was the most frequently applied (*N* = 6), followed by the Theory of Value Co-Creation and Pine and Gilmore's (1999) Experience Economy Model (*N* = 3). Despite evidence of embryonic theoretical maturity, there was limited consistency or saturation in theoretical approaches applied, with the selection of theories, models, and frameworks not clustered within a specific discipline and demonstrating a fragmented approach to knowledge creation. For instance, 37 out of 40 theories, models and frameworks were only applied two times or less, apart from the top three theories, models, and frameworks, demonstrating a stagnation in the ability to stimulate constructive conversations designed to advance knowledge on TMVEs.

**Table 6:** Top three frequently applied theories, models, and frameworks in TMVEs

Theories/Models/Frameworks	Frequency	%
Technology Acceptance Model	6	11.32
The Theory of Value Co-creation	3	5.66
Pine and Gilmore's (1999) Experience Economy Model	3	5.66
Total	12	22.64

Types of ICT were coded according to Flavián et al. (2019), which proposed an “EPI Cube”, which classified the technologies into categories. The EPI cube was established based on three dimensions – “technological embodiment”, “perceptual presence”, and “behavioral interactivity”. Each dimension of the EPI cube comes with complex divisions based on subtle differences of technologies (Flavián et al. 2019). For instance, it segmented Head-Mounted Displays (HMD) to Augmented Reality (AR) HMD, Virtual Reality (VR) HMD, and Mixed Reality (MR) HMD. However, a selection of studies in the review did not state explicitly the fine-grained distinctions between technologies (Amitrano et al. 2018; Kéfi and Pallud 2011; Kuflik et al. 2015; Kuksa and Tuck 2011; Pallud and Monod 2010; Sylaiou et al. 2010), thus, a classification was made according to a list of technologies classified by Ch’ng et al. (2019) and Mohd Noor Shah and Ghazali (2018) which were based on the functionality of technologies. Drawing on this analytical framework, five types of technology were displayed in Table 7.

**Table 7:** 5 types and forms of technology utilised in TMVEs

Types of technology	Frequency	1998–2004	2005–2010	2011–2016	2017–2022
Smart technologies	68		4	10	54
Mobile communication	29		5	10	14
Virtual communities	24	1	1	4	18
Computer displays	15	1	4	2	8
Social media	13			1	12

Smart technologies dominated existing studies on TMVEs, with 13 types of smart technologies, such as AR (*N* = 19) and VR (*N* = 14) emerging as topical especially since 2017. Other smart technologies such as MR, Eye Tracking, and 3D printing were becoming embedded in contemporary work on TMVEs. Other classifications according to the EPI cube, such as mobile communication (*N* = 29), virtual communities (*N* = 24), and social media studies (*N* = 13) have also gained momentum particularly since 2011. Studies on mobile guides (*N* = 24) represented a majority of research under the classification of mobile communication. Studies on virtual communities used to focus on traditional types of technology, such as websites (*N* = 14), but have evolved to adapt to technological change inspired by the advent of Metaverse (*N* = 2). Research foci on social media has expanded, especially in the last five years, with Twitter, Instagram, and Facebook becoming increasing subjects of investigation. Computer displays included external devices, such as multimedia applications and information kiosks, which involved a low degree of behavioural interactivity, demonstrating limited growth in research in this specific classification. Table 8 provides further explanation of some technological terms classified under different types of technology and their application in the reviewed articles.

**Table 8:** Technological terms and their application in the dataset under the types of technology

Types of technology	Explanation	Examples
*Smart technologies*		
VR	It is a computer-generated simulation of an environment that can be experienced through audio, visual, and tactile sensations. Its goal is to create a lifelike and immersive experience that can transport the user to a different world or location	Sylaiou et al. (2010)
AR	Different from VR, AR is typically viewed through a device, such as a smartphone or a tablet, that uses its camera to capture the surrounding environment and then superimposes the digital content onto it	Chang et al. (2014)
MR	It is a technology that blends both VR and AR. It allows digital objects to be integrated into the real world and interact with it in real-time, providing users with an immersive experience of both virtual and real-world environments	Nisi et al. (2018)
Eye Tracking	It is the act of monitoring and documenting the motion of an individual's eyes as they observe visual information	Pelowski et al. (2018)
3D printing	It is a way of making objects by building them up layer by layer using a special machine	Karaduman et al. (2022)
*Mobile communication*		
Mobile guides	Mobile guides are applications designed for mobile devices such as smartphones and tablets that provide users with information and guidance on a particular topic or location	Dou et al. (2020)
*Virtual communities*		
Metaverse	The Metaverse is a virtual world or universe where people can interact with a computer-generated environment and other users through avatars or digital representations of themselves	Lee et al. (2022)
*Computer displays*		
Multimedia applications	They are computer programs that use different types of media, like pictures, sounds, videos, and words, to show information or provide fun experiences to people using the computer	Economou (1998)

As illustrated in Table 9, the majority of studies adopted quantitative methods, with exponential growth in this approach since 2017. In contrast, qualitative approaches, while increasingly moving, were only applied in 28 out of 122 articles. Scholars were increasingly adopting a mixed methods approach for studies on TMVEs, with marked increase between 2017 and 2022.

**Table 9:** The progress of methods and approaches adopted over time

Approaches	No. of studies	1998–2004	2005–2010	2011–2016	2017–2022
Quantitative	54	1	1	6	46
Qualitative	28		3	8	17
Mixed methods	31	1	3	4	23
Conceptual	9				

As displayed in Table 10, studies had a tendency to apply more than one data collection technique, with an average of 1.52 tools applied across each of the 122 studies. While traditional data collection techniques such as questionnaires, semi-structured interviews, and participant observation were prevalent in existing studies, a diverse range of data collection techniques were emerging in prevalence. In addition to surveys, interviews, and observations, experimental designs were emerging, consisting of physiological techniques, such as Eye Tracking which captured museum visitors’ attention. Other data techniques, such as secondary data and website review have been utilised to understand visitors’ museum experience through online storytelling. Contemporary techniques such as netnography, shadowing, and personal meaning mapping were emerging, to provide more depth to the contributions offered by traditional data collection tools, with six studies emerged in research.

**Table 10:** Data collection techniques applied in TMVEs

Data collection techniques	Frequency
Questionnaire/Survey	74
Semi-structured interview	35
Observation	28
Experiment	14
Eye Tracking	6
Secondary data/Website review data	12
Focus group	7
Ethnography	4
Quasi-experiment	3
Shadowing	3
Personal meaning mapping	2
Auto-ethnography	1
Netnography	1
Expert review	1
Not specified	14

## Discussion

This research systematically assessed the emerging body of work focused on TMVEs, explicitly examining trends of publication volume in research across time, clustering within journals, the geographic locations of fieldwork, progress in concepts, theories, models, and frameworks, the evolution in the types and forms of technology, and innovation in data collection techniques applied. In evaluating the achievement to address four research objectives, this study found research on TMVEs was initially slow to evolve, though has experienced exponential growth since 2017. Discourse across time was not concentrated in a distinct cluster of journals, though was spread across a number of publication outlets, with a couple of dedicated journals including *Museum Management and Curatorship* and *Current Issues in Tourism* providing a small concentration of published literature. The limited research contribution in significant journals as mentioned previously highlights the need for future research endeavour on publishing in high-quality, theoretically informed and methodologically sound research in journals to better communicate the potential of museums. Notably, a substantial proportion of reviewed articles conducted research in Europe, which is where a high proportion of museums are located, particularly around Western Europe such as Italy (UNESCO 2020). While the findings of this paper showed that Europe had the largest number of fieldwork conducted, it is recommended for future research to consider if geographic distribution of fieldwork locations strongly correlates to the number of museums they have in the destination. Nonetheless, the significant number of museums located in the United Stated which presents an opportunity to dedicate more scholarly attention, along with a dearth of studies undertaken in Australia, New Zealand, and South Africa, with such destinations offering potential opportunities for comparative case studies.

The concepts studied in early research towards recent literature experienced an evolution in the depth of focus. For instance, early studies on learning focused on the learning effects of technology on visitors in the museum (Economou 1998), slowly transitioning to educational outcomes of field trips among primary, high school, and university students (Charitonos et al. 2012; Sung et al. 2010a, b; Yoon et al. 2012). However, following 2016 studies that were interested in the concept of learning, began to focus on issues such as the role of technology in “immersive learning” explicitly examining the ability of visitors to acquire additional knowledge through engagement in TMVEs (Hughes and Moscardo 2017; Lo et al. 2019). Related concepts such as experience and interaction also had a considerable amount of research attention, with a fundamental evolution in how these concepts were evident in the body of knowledge. For instance, early studies tended to investigate onsite visitor experiences in museums (Dirk vom and Christian 2005; Economou 1998; Sung et al. 2010a, b). However, articles began to explore the critical role of technology across other stages of the visitor experiences, with an emphasis on how technology can enhance the recollection stage (Jarrier and Bourgeon-Renault 2012; Kuflik et al. 2015). While “immersive learning” has gained traction in recent studies, limited focus is on how the immersion of tourists impacts on their satisfaction in smart museums (Li et al. 2023).

Interaction focused on social interactions between visitors in museums inspired by technology (Dirk vom and Christian 2005; Heath and Vom Lehn 2008), with sustained research focus on this area transitioning to the role of technology in the decline of human interaction (Ponsignon and Derbaix 2020). Intention is also a recurring topic, however, studies predominantly focused on topics such as revisit intentions (Lee et al. 2020; Yang and Zhang 2022), intentions to purchase of museum souvenirs (Dou et al. 2020), intentions to financially support museums (Zhang et al. 2022; Zollo et al. 2022), and intentions to use future technology (Carvajal-Trujillo et al. 2021). Limited studies have sought to explore the critical role of technology for intentions to perform pro-environmental and pro-social behaviours in response to museum visitation. For example, Wheaton et al. (2016) noted the potential of technology motivated pro-environmental action after a nature-based tourism experience. Engagement with this literature could open up future avenues of research focused on how the integration of technology in museums can facilitate environmental or social behavioural intentions among visitors. In addition, while there is foundational work which examines the influence of museum mobile app on the intention to purchase museum souvenirs, future research could consider the internal mechanisms of consumers’ purchase intention of museum souvenirs by aligning tourists' perceived image of the souvenirs with souvenir brand identity facilitated by technology (Guo and Zhu 2023).

Perceived ease of use, embedded in the Technology Acceptance Model (TAM) (Davis 1989), was also an issue raised in existing studies (Goel et al. 2022). Such articles had a tendency to assess how difficult it is for blind or visually impaired people to use beacons—as a way-finding and guiding system in museums (Landau et al. 2005). However, studies are yet to explore perceived usefulness of technology, as recommended by Davis (1989), as perceived usefulness potentially has a high correlation with the user acceptance. For instance, perceived usefulness of technology has provided critical insights in the conceptually related fields of online learning and online purchasing (Kucukusta et al. 2015; Saade 2007; ThaeMin and JongKun 2005), with Kucukusta et al. (2015) and Saade (2007) noting that perceived usefulness is influential in the formation of behavioural intentions.

While there was limited in-depth engagement with a single theoretical perspective TAM was most frequently utilised in studies on smart tourism (Dorcic et al. 2019). All of six reviewed articles modified TAM, including extended variables added for testing. For example, Kang et al. (2018) utilised TAM by adding perceived interactivity and enjoyment to test visitors’ satisfaction toward the museum experience. The Theory of Value Co-creation and Pine and Gilmore's (1999) Experience Economy Model were adopted as well, though a limited concentration of discourse around these models. More in-depth engagement with experience economy presents an opportunity to connect with embedded concepts such as value co-creation. For example, a study by Neuhofer et al. (2013) proposed a “four-quadrant Tourism Experience Value Matrix” which applied an experience economy perspective to better understand how technology can co-create value in experiences. Consequently, adding depth to current perspectives offered, as well as broadening engagement with alternative theoretical perspectives, present an opportunity investigating the underlying psychological factors influencing museum visitor experiences aided by technology.

Emerging perspectives, such as Mindfulness Theory and Flow Theory offer substantial opportunities to explore whether technology contributes to deep engagement of museum visitors in experiences (Hughes and Moscardo 2017; Zhang and Abd Rahman 2022). Mindfulness Theory and Flow Theory explain the psychological aspects of tourist experiences, which capture attention and elicit emotion, which in turn could be leveraged to target the behavioural intentions of museum visitors (Frauman and Norman 2004). There are two studies in the dataset that employed Mindfulness Theory and Flow Theory, of which mindful tourists and visitors who experienced flow have an in-depth understanding of and seamless interaction with the environment in the smart museum (Hughes and Moscardo 2017; Zhang and Abd Rahman 2022). Subsequently, Mindfulness Theory and Flow Theory can be utilised to improve theoretical engagement in future studies on TMVEs. Emergent perspectives such as Mental Imagery Theory and Attentional Control Theory were applied in one of the reviewed articles (He et al. 2018). These conceptually related perspectives could be considered to delve into how and why different design elements of technology can influence the mental imagery of museums perceived, captured, and stored in autobiographical memory (Skavronskaya et al. 2017). Such approaches can broaden perspectives on how visitors perceive things differently through investigating the mental processes triggered between stimuli and behaviour (Skavronskaya et al. 2020).

De Angeli et al. (2020) questioned the traditional data collection techniques used to evaluate museum visitors’ emotions and proposed the emoji-based survey can elicit a more precise indication of emotions that visitor feel during the experiences. However, while this represented the beginning of an emergent debate on contemporary data collection techniques, Eghbal-Azar and Widlok (2013) asserted that Eye Tracking technique held potential for visitors “appropriating exhibitions”. Nonetheless, there is a dearth of research which currently adopts physiological techniques such as Eye Tracking. Recent studies in tourism have demonstrated an emergence of studies which examine visitors’ attention using Eye Tracking technique (Li et al. 2022; Scott et al. 2019). Eye-tracking presents an opportunity to objectively measure how visitors perceive and pay attention to exhibitions and associated interpretative content facilitated by technology. Cognitive Appraisal Theory (CAT) has strong potential to provide sound theoretically grounding to the adoption of cutting-edge physiological data collection techniques (e.g., Eye-tracking) which can stimulate constructive discourse on how the technological integration into museums affects virtual and real museum experiences (Liu et al. 2023). CAT, drawn from the cognitive psychology, has emerged recently in tourism studies to explain why a certain emotion is felt by tourists when experiencing activities (see examples Liu et al. 2022; Scott 2020). In the smart museum, the application of Eye Tracking could be to capture visitors’ interests or preferences, with the theoretical underpinning of CAT for a better understanding of why visitors are interested in certain exhibits through appraising different emotions felt by visitors (Ma et al. 2013). For example, a visitor may appraise an exhibit as being interesting and informative, leading to a positive emotional response. Alternatively, the visitor may appraise the exhibit as being dull or unengaging, leading to a negative emotional response. In so doing, the effectiveness of designing experiences with the technology integration in museums is evident.

**Fig. 4:**
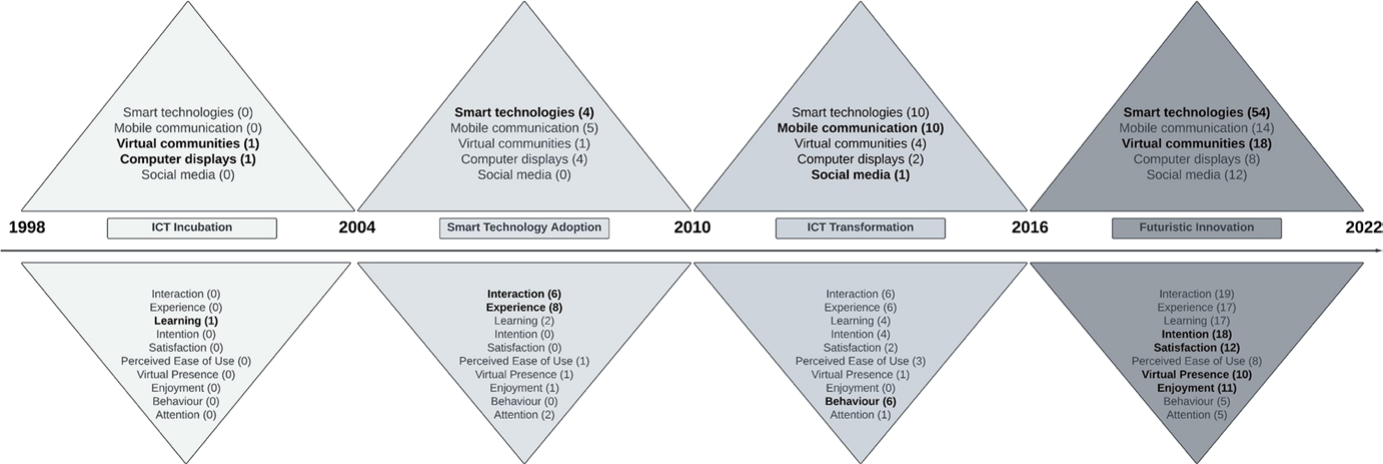
A Four Stage Model of Evolution

While the body of knowledge on TMVEs is arguably fragmented, this research revealed the emergence of four sequential stages which depict an evolution in the types of technology studied, juxtaposed with the concepts prevalent in existing discourse. Figure 4 presents a Four Stage Model of Evolution which integrates the types of technology with the concepts studied.

As displayed in Fig. 4, research on TMVEs can be synthesised into four key stages of ICT Incubation (1998–2004), Smart Technology Adoption (2005–2010), ICT Transformation (2011–2016) and Futuristic Innovation (2017–present). Elements in each triangle were highlighted in bold to differentiate four stages. Stage one entitled ICT Incubation reflects the integration of basic ICT applications, such as multimedia application and museum website and limited conceptual engagement. Stage two marks the adoption of studies on smart technologies, such as VR and AR, which were introduced into the literature, and as a consequence conceptual engagement broadened to include a deeper focus on interaction and aspects of the experience. Stage three refers to a period which assessed an increasingly diverse range of technologies, signifying an ICT transformation in museums. Different classifications of technologies emerged during this period, ranging from computer displays (multimedia application) to smart technologies (Radio Frequency Identification), with conceptual engagement deepening to include studies on behaviour. Stage four reflects exponential growth in research on TMVEs, with an emphasis on future innovations such as MR, Eye Tracking, 3D printing, and Metaverse, emerging in discourse designed to enhance visitor experiences in museums, with detailed conceptual engagement with emergent concepts such as presence, enjoyment, satisfaction, and intention adding to the knowledge on more established concepts such as learning, experience, and interaction.

The emergence of innovative technologies in the museum experiences, such as VR, AR, MR, and Metaverse as reflected in the model is gaining research traction (Rudi 2021; Yang and Zhang 2022). For example, Verhulst et al. (2021) claimed that VR and AR could lead to different immersive visitor experiences. Similarly, Hammady et al. (2021) introduced a MR virtual guide serving as an interpretation role for visitors to present museum information and storytelling effectively. Despite this, limited studies have focused on how these emerging technologies can facilitate museums to be an accessible and inclusive place for diverse visitors, such as people with disabilities and senior populations. As the increasing number of consumers with disabilities emerged in the tourist market, VR, AR, MR, and Metaverse have the potential to provide virtual museum experiences for them without the need to visit the physical museum (Tlili et al. 2021). In addition, it is highly recommended that future discourse to critically examine how different segments of visitors engage in TMVEs (Brochado et al. 2022). In so doing, positive marketing outcomes can be achieved by designing tailored technology-related strategies for each desired market segment (Tanti and Buhalis 2017; Volchek et al. 2019).

## Conclusions

This research critically examined the evolution of research on TMVEs. Specifically, this study synthesised 122 eligible articles for review, and coded and analysed the trends of research on TMVEs, exploring journal concentrations and the fieldwork conducted. Importantly, the study unveiled the progress in concepts, theories, models, and frameworks during the last 34 years, categorised the types and forms of technology discussed on TMVEs, and examined the methods and data collection techniques progressed over time.

Three major findings are highlighted from this systematic review. First, the high concentration of studies conducted in Europe illustrates the popularity and maturity of technology-integrated museum development. Museums such as in Italy were prevalent, so there is a requirement for comparative studies on other geographic and cultural contexts. Second, limited theoretical engagement in existing discourse reflects the need for more in-depth theoretical frameworks to be embedded in future research on TMVEs. Mindfulness Theory, Flow Theory, and Cognitive Appraisal Theory could be considered associated with the applications of innovative data collection techniques such as Eye Tracking to solve theoretically informed research problems. Critical attention could be paid to how the role of technology in museum visitor experiences stimulates complex mental processes which modify behaviour and stimulate memory. Third, there are several classifications of technology in tourism, ranging from the categorisation on the basis of the intensity of technology interactivity (Neuhofer et al. 2014), functionalities of technology (Ch’ng et al. 2019; Mohd Noor Shah and Ghazali 2018), to complex dimensions of technology subdivisions (Flavián et al. 2019). However, limited scholarly attention has been paid to the types and forms of technology in the museum sector. In this vein, five categories—smart technologies, mobile communication, virtual communities, computer displays, and social media were utilised to classify existing studies according to Ch’ng et al. (2019), Flavián et al. (2019), and Mohd Noor Shah and Ghazali (2018).

Drawing on this approach, this research develops a Four Stage Model of Evolution developed to depict four sequential time phases which provide an overview of the progress in research on TMVEs, specifically, ICT Incubation (1998–2004), Smart Technology Adoption (2005–2010), ICT Transformation (2011–2016), and Futuristic Innovation (2017–2022). This model integrates the evolution of different types of technology and concepts prevalent in studies over time and can be utilised to inform future scholarship in the area. The Four Stage Model of Evolution presents an evolution of how concepts have shifted across time, designed to stimulate further discourse. Furthermore, this model contributes to the clarity of conceptual ambiguity of technology development in the museum context, with the five types of technology being the first established according to three technology categorisations mentioned previously. Future research should explore the fourth stage of Four Stage Model of Evolution—Futuristic Innovation, with Metaverse having the potential of transforming museum visitor experiences in the future (Buhalis et al. 2022).

Outcomes of this research are designed to provoke insights for the museum management and curators interested in how technological innovation can enhance the visitor experiences. With the exponential growth of technology utilisation into museums, the systematic review draws a comprehensive picture of what has been investigated and what might be taken into consideration in future avenue for museum curators, designers, and practitioners to consider what technology is worthy of investment and how to improve visitors’ experiences with different types of technology. In this vein, the financial burdens of museums could be relieved by choosing the suitable technology to boost admissions among existing visitors and attract new visitor markets by providing a more in-depth engaging experience for museum visitors in our rapidly transforming twenty-first century society (He et al. 2018). Specifically, the technological innovations that have surfaced in the fourth stage of the model, including but not limited to MR and Metaverse, hold considerable promise for museum managers and curators. These advancements are particularly pertinent in light of the pressing need to engage new customer segments, such as Generation Z, born between the mid-1990s and early 2010s (Buhalis and Karatay 2022; Buhalis et al. 2023).

The current study has limitations, which were minimised or mitigated through the research process. First, as common in existing tourism literature the SQLR only selected English papers. However, as the majority of reviewed articles were conducted in Europe, there might be a considerable number of studies published in French, Italian, and Spanish. Due to the linguistic background limitation of the researchers, it might not guarantee the comprehensiveness of studies coverage on TMVEs and future comparative studies on other languages would further add validation to the content in this review. Second, although the reproducibility of SQLR is important, the coding and analysis were subject to the researchers’ interpretations (Le et al. 2019), however techniques such as expert review were integrated to minimise researcher bias. Lastly, the keywords selection was based on literature and expert review process, however, new terms of technology might be generated as time passes, as technology development is rapid and unpredictable.
